# Risk factors for mild cognitive impairment in type 2 diabetes: a systematic review and meta-analysis

**DOI:** 10.3389/fendo.2025.1617248

**Published:** 2025-06-16

**Authors:** Yi Zhao, Hanlin Wang, Guanghao Tang, Leilei Wang, Xuewen Tian, Ran Li

**Affiliations:** ^1^ School of Postgraduate Education, Shandong Sport University, Jinan, Shandong, China; ^2^ School of Life Sciences, Ludong University, Yantai, Shandong, China; ^3^ School of Exercise and Health, Shandong Sport University, Jinan, Shandong, China

**Keywords:** mild cognitive impairment, type 2 diabetes, risk factors, diabetes duration, HbA1c, meta-analysis

## Abstract

**Background:**

Mild Cognitive Impairment (MCI), a transition between normal aging and dementia, is linked to higher dementia risk and potential reversibility. Type 2 Diabetes Mellitus (T2DM), affecting over 537 million adults worldwide, increases susceptibility to MCI, with higher cognitive decline prevalence in diabetic populations. Previous meta-analyses focused on isolated factors, neglecting multidimensional interactions. This study synthesizes T2DM-MCI risk factors across clinical, lifestyle, and biochemical dimensions to support early identification and intervention of cognitive dysfunction in T2DM populations.

**Materials and Methods:**

This systematic review and meta-analysis, following PRISMA guidelines, searched five databases for articles published from January 1, 2014, to December 31, 2024. Studies were screened based on predefined criteria, with data extracted independently by two researchers. Quality was assessed using Newcastle-Ottawa Scale (NOS) and Joanna Briggs Institute (JBI) tools. Data were analyzed using RevMan software, with odds ratio (OR) and 95% CI as effect size measures. Heterogeneity was assessed using I² statistics, and subgroup analyses were conducted for factors with ≥10 studies.

**Results:**

30 studies with 10,469 participants were included. Prevalence rate of MCI in T2DM was 44.1%. Significant associations were found between T2DM-MCI and age (OR = 1.06, P = 0.01), female sex (OR = 1.23, P = 0.05), diabetes duration (OR = 1.07, P = 0.03), education (OR = 0.82, P = 0.0001), smoking (OR = 1.44, P = 0.003), hypertension (OR = 2.25, P < 0.001), cardiovascular disease (CVD) (OR = 2.61, P < 0.001), glycated hemoglobin (HbA1c) (OR = 1.33, P = 0.001), and homeostasis model assessment of insulin resistance (HOMA-IR) (OR = 1.95, P = 0.02).

**Conclusion:**

This meta-analysis identifies advanced age (≥60 years), female sex, prolonged Diabetes duration (8–9 years), elevated HbA1c (>9%), and low education (≤6 years) as key predictors of MCI in T2DM, with significant dose-response relationships. Vascular comorbidities, insulin resistance, and inflammatory markers further exacerbate risks. Clinical priorities include rigorous glycemic control (HbA1c <7%), targeted cognitive screening for high-risk subgroups, and multidisciplinary care for patients with microvascular complications. However most of the studies included in this study come from Chinese people, so the generalization of the results may be limited.

**Systematic review registration:**

https://www.crd.york.ac.uk/prospero, identifier CRD420250637336.

## 1 Introduction

Mild Cognitive Impairment (MCI) represents a transitional phase between normal cognitive aging and dementia, and it is potentially reversible in nature (1). Individuals with MCI exhibit significantly elevated risk of progressing dementia compared to cognitively healthy populations (2). Type 2 Diabetes Mellitus (T2DM), one of the most prevalent metabolic disorders globally, continues to show escalating prevalence rates. According to International Diabetes Federation (IDF), the global adult population with diabetes exceeded 537 million in 2021, over 90% of whom had T2DM, and this figure is projected to surpass 783 million by 2045 (3). Concurrently, cognitive health concerns in T2DM patients, particularly MCI comorbidity, have gained increasing attention. Studies indicated that T2DM not only serves as a risk factor for MCI but also accelerates its progression to dementia (4). Diabetic individuals have a 1.25 to 1.91 times higher likelihood of developing cognitive impairment than non-diabetic individuals (5). Epidemiological data suggest that the prevalence of MCI among T2DM patients ranges from 19.9% to 45.0% (6, 7).

MCI manifests through impairments in core cognitive domains, including memory (8) and executive function (9), and is associated with multiple adverse clinical outcomes. For instance, a meta-analysis revealed that older diabetic patients with comorbid MCI face a higher risk of falling (10). Assessments using the World Health Organization Quality of Life Assessment for Older Adults further demonstrated significantly reduced quality-of-life scores among MCI patients across dimensions such as autonomy, engagement in past/present activities, and social participation (11). These findings collectively highlight that MCI not only serves as an early indicator of cerebral functional decline in T2DM patients but also exacerbates the disease burden through multiple pathways.

Early intervention for MCI in T2DM patients is therefore critical for preserving cognitive function and preventing dementia. The pathophysiological mechanisms underlying T2DM-MCI comorbidity involve complex interactions: chronic hyperglycemia directly impairs cognitive function through the deposition of advanced glycation end-products (12, 13), blood-brain barrier disruption (14, 15), and hippocampal neuronal apoptosis (16, 17). Notably, the MCI stage represents a reversible therapeutic window (18, 19). Meta-analyses indicate that early identification and control of risk factors significantly reduce dementia conversion risks and subsequent healthcare expenditures (20). Comprehensive management strategies could potentially prevent or delay up to 40% of dementia cases (21), underscoring the need for proactive preventive measures.

Despite established evidence on T2DM-MCI determinants, controversies persist regarding the heterogeneity of factors and their relative contributions. Existing meta-analyses predominantly focus on isolated factors, such as glycated hemoglobin (HbA1c) levels (22),or diabetes duration (23), lacking systematic integration of multidimensional elements, including demographic/clinical characteristics, biochemical parameters, lifestyle factors, and disease management. For example, while some studies have reported significant associations between smoking history (24, 25) and T2DM-MCI risk, others have fail to corroborate this relationship (26, 27). Similarly, conflicting evidence exists fasting plasma glucose (FPG) and HbA1c, with some studies finding no significant association (28, 29) and others reporting clear links (30, 31). To address these inconsistencies, the present study conducts a meta-analysis to consolidate current evidence, systematically evaluating risk factors and their weighted contributions across four dimensions: demographic and clinical characteristics (including age, sex, and diabetes duration), lifestyle factors (smoking and alcohol consumption), disease management (hypertension and depression), and biochemical indicators (HbA1c). The aim is to provide evidence-based support for early identification and intervention of cognitive dysfunction in T2DM populations.

## 2 Methods

This study was conducted as a systematic review and meta-analysis, with the study protocol prospectively registered in the PROSPERO database (Registration ID: CRD420250637336). The methodology strictly adheres to the 2020 Preferred Reporting Items for Systematic Reviews and Meta-Analyses (PRISMA) guidelines (32). Institutional ethics committee approval was waived due to the exclusive use of aggregated data from previously published studies.

### 2.1 Search strategy

As of December 31, 2024, we systematically searched five databases (PubMed, Embase, Web of Science, Google Scholar, and Elsevier) for articles published from January 1, 2014, to December 31, 2024. The search strategy combined the following terms using Boolean operators: (Diabetes Mellitus, Type 2 OR T2DM OR type 2 diabetes) AND (Cognitive Dysfunction OR Mild Cognitive Impairment OR MCI) AND (risk factors OR predictors OR determinants). In addition, manual searches of reference lists from identified articles and relevant reviews were performed to supplement the electronic search.

### 2.2 Study selection

One researcher (H.L.W.) performed the initial literature search and removed duplicates. Two researchers (Y.Z. and G.H.T.) independently screened titles and abstracts against predefined inclusion and exclusion criteria. Full texts were retrieved if either reviewer deemed an article potentially eligible. The reviewers then independently assessed the full-text articles for final inclusion. Discrepancies were resolved through consultation with the corresponding author (R.L.).

The inclusion criteria were as follows (1): study involving patients diagnosed with T2DM and MCI (2); case-control, cohort, or cross-sectional designs (3); data convertible to odds ratio (OR) with a 95% confidence interval (CI) (4); reporting at least one risk factor (5); use of multivariable logistic regression to identify T2DM-MCI determinants; and (6) clear diagnostic criteria for MCI. The exclusion criteria were (1): duplicate publications (2); reviews, letters, or non-research articles; and (3) non-English publications.

### 2.3 Data extraction

Data extraction was independently performed by two investigators (Y.Z. and L.L.W.) using standardized forms. The following parameters were recorded: first author’s name, mean age, sex distribution, publication year, study location, sample size, prevalence of T2DM-MCI comorbidity, and reported risk factors. Quantitative measures, including OR with corresponding 95%CI, were extracted for each determinant. The extracted variables were stratified into four etiological domains: 1) Demographic and Clinical Characteristics: age, sex, diabetes duration, body mass index (BMI), and educational attainment. 2) Lifestyle Factors: alcohol consumption, smoking status. 3) Comorbidity Management: depression, hypertension, cardiovascular disease (CVD), and diabetic retinopathy (DR). 4) Biochemical Indicators: HbA1c, homeostasis model assessment of insulin resistance (HOMA-IR), low-density lipoprotein cholesterol (LDL-C), high-sensitivity C-reactive protein (HS-CRP), FPG, and high-density lipoprotein (HDL).

### 2.4 Quality assessment

Methodological quality was assessed using the Newcastle-Ottawa Scale (NOS) for cohort and case-control studies (33) and the Joanna Briggs Institute (JBI) Critical Appraisal Tool for cross-sectional studies (34). The NOS evaluates three domains: selection, comparability, and exposure/outcome ascertainment, with a maximum score of 9 points. Studies scoring ≤ 5 points were classified as low quality, scoring between 5–7 points were classified as medium quality, and scoring > 8 were classified as high quality. The JBI tool employs a percentage-based scoring system, with a maximum score of 8 points, categorizing studies as high quality (≥ 7), moderate quality (5, 6), or low quality (≤4). Only studies meeting quality thresholds (NOS ≥ 5 or JBI ≥5) were retained. Two investigators (G.H.T. and H.L.W.) independently conducted quality assessments. Discrepancies in scoring were resolved through consultation with the corresponding author (R.L.).

### 2.5 Data analysis

All statistical analyses were performed using Review Manager (RevMan) software, version 5.4. OR with corresponding 95% CI served as effect size measures. Heterogeneity was quantified using I² statistics and P-values, with thresholds set at P < 0.1 or I² > 50% indicating substantial heterogeneity. The fixed-effects model assumes consistent effect sizes across studies, suitable for low heterogeneity (I² ≤50%) and calculates pooled effect size through weighted averages. The random-effects model assumes variability in effect sizes and is used when significant heterogeneity exists (I² >50%), incorporating study differences through weighted averages. Therefore, a random-effects model is chosen when I² >50%, and a fixed-effects model is used otherwise. Subgroup analyses were conducted for factors with ≥ 10 studies. Sensitivity analyses were performed by switching between fixed-effects and random-effects models for outcomes demonstrating I² > 50%. Publication bias was assessed through funnel plot symmetry evaluation and Egger’s linear regression test, which was restricted to factors with ≥10 studies. A significance level of P < 0.05 was defined for all inferential analyses.

## 3 Results

### 3.1 Search results

**Figure 1:**
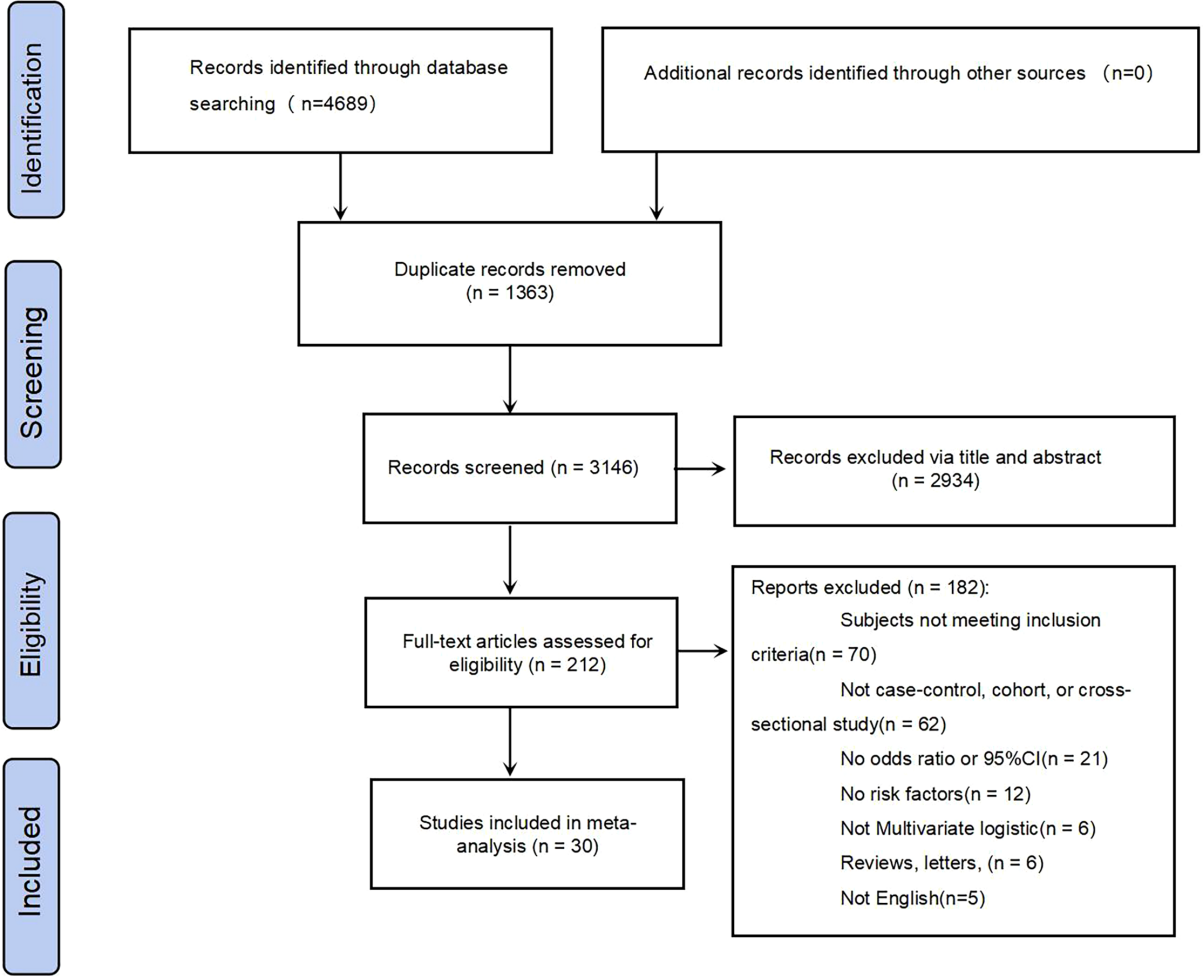
Flow chart of PRISMA selected for the study.

The systematic search initially identified 4,689 citations. Following duplicate removal, 3,326 records were subjected to preliminary screening. Title/abstract screening excluded 2,956 non-eligible studies, leaving 212 articles for full-text assessment. Ultimately, 30 studies met the inclusion criteria and were included in the meta-analysis of T2DM-MCI determinants. The complete screening protocol is presented in the PRISMA flowchart (**Figure 1**).

### 3.2 Study characteristics

**Table 1** summarizes the characteristics of the included studies. The pooled analysis comprised 30 studies conducted across five countries, involving 10,469 participants with T2DM, including 4,516 cases with comorbid MCI (prevalence rate = 44.1%). These investigations, published between 2014 and 2024, had sample sizes ranging from 103 to 1,278 participants. The mean age of the study populations ranged from 50 to 84 years, with female participants representing 41.8% of the total cohort.

**Table 1:** Study characteristics.

Inclusion Of Studies	Year of Publication	Country	Number of cases		MCI Diagnostic basis	Factors
			Case Group	Control Group		
Malgorzata Gorska-Ciebiada (45)	2014	Polish	87	189	C G	⑦⑨⑩⑬⑯
San-Shan Xia (46)	2020	China	174	76	E G H	⑤⑫⑬
Malgorzata Gorska-Ciebiada (44)	2016	Polish	62	132	C G	⑦⑨⑬
Arpita Chakraborty (6)	2021	India	458	820	A G	①②③⑦⑬⑯
Dan Guo (26)	2019	China	57	69	C G H	⑬
Yun Jeong Lee (48)	2014	Korea	74	152	A G	①③⑦⑫⑱
Yuanyuan Jiang (47)	2024	China	186	120	A G	②③④⑦⑬
Wei (35)	2024	China	40	208	B H	①
Tao Luo (49)	2024	China	50	53	E G	⑭
Minli Liu (36)	2024	China	125	183	F H	②③⑦
Hui Zhang (51)	2023	China	58	85	C G	①③⑬⑭
Sai Tian (50)	2018	China	94	108	C G	⑬⑭
Hongjun Zhao (53)	2019	China	48	30	A G	⑬⑭⑮
Haoqiang Zhang (52)	2021	China	235	262	C G	①②⑦⑫
Wei Li (37)	2019	China	56	200	B G H	④⑦
Zhichun Sun (54)	2018	China	151	564	A G	①③⑦⑧⑬
Miaoyan Zheng (39)	2019	China	63	63	A H	⑪
Fanyuan Ma (38)	2023	China	280	224	A G	⑦
Yaoshuang Li (55)	2024	China	204	320	A G H	①⑦⑧⑨
Jie Sun (56)	2016	China	75	71	C G	⑬
Li Ma (40)	2024	China	94	242	A G H	①⑤⑥⑦⑧⑩⑬⑱
Xuewei Tong (57)	2023	China	313	303	E G	①③⑤⑥⑦⑧⑱
Haina Zhang (27)	2023	China	91	201	C G	③⑦⑭
Ruonan Gao (41)	2024	China	44	31	F G	①③⑫⑯⑰
Xueyan Liu (60)	2024	China	451	447	A G	①②③④⑤⑦⑱
Malgorzata Gorska-Ciebiada (43)	2020	China	62	132	A G	⑦⑨⑩⑪
Johanda Damanik (58)	2019	China	47	50	A G	⑧
Jingcheng Ding (59)	2023	China	112	110	A G H	①⑥⑬⑮
Lina Ma (61)	2017	China	100	112	A G H	⑦⑧⑭
Yuxia Gao (42)	2016	China	690	287	B G	③⑥⑬⑮⑯

Diagnostic criteria for MCI were categorized as follows: A: Mental Status Examination, B: 1999 Petersen criteria, C: Diagnostic protocol proposed by the European Alzheimer’s Disease Consortium MCI Working Group (2006), D: 2001 Petersen criteria, E: Core Clinical Criteria for Dementia Diagnosis by the National Institute on Aging-Alzheimer’s Association, F: Chinese Guidelines for Diagnosis and Treatment of Dementia and Cognitive Impairment, G: Montreal Cognitive Assessment, H: Mini-Mental State Examination.

Factors:① Age ② Female ③ Diabetes duration ④ Depression ⑤ Alcohol ⑥ Smoking ⑦ Educational attainment ⑧ BMI ⑨ CVD ⑩ Hypertension ⑪ Hs-CRP ⑫ LDLC ⑬ HbA1c ⑭ HOMA-IR ⑮ FPG ⑯HDL ⑰ DR ⑱ Male.

### 3.3 Methodological quality assessment、sensitivity analyses and publication bias assessment

**Figure 2:**
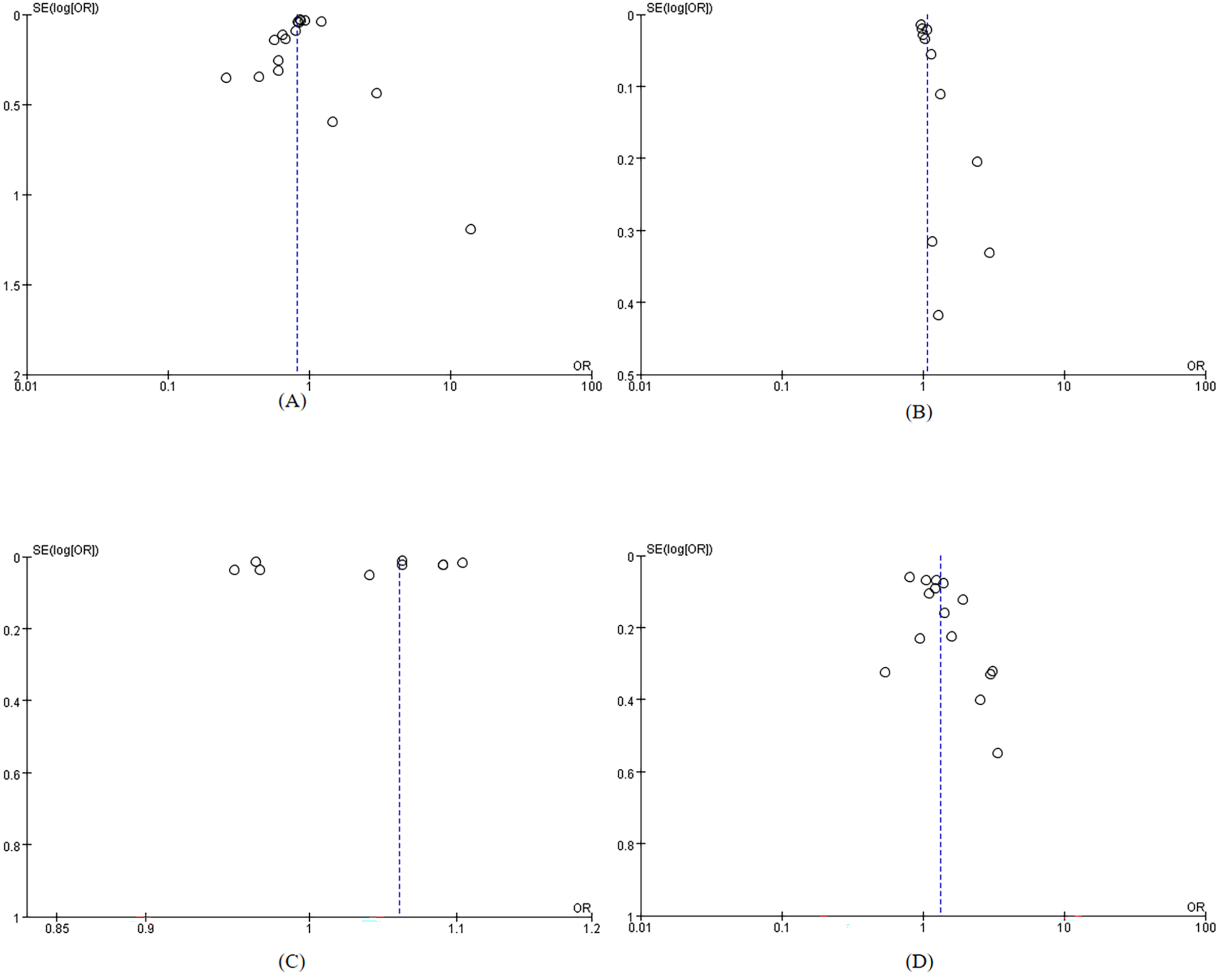
Funnel plot **(A)**Level of education; **(B)** Duration; **(C)** Age; **(D)** HbA1c.

The included studies were of moderate to high quality. Overall quality assessment showed that 8 articles (35–42) were classified as high quality, while 22 articles (6, 26, 27, 43–61) were categorized as medium quality, For specific scores, see **Supplementary Table S1** and **Supplementary Table S2**. The included studies demonstrated stability in sensitivity analyses. Funnel plots indicated no significant publication bias (**Figure 2**). **Table 2** presents the results of Egger’s test, confirming no substantial publication bias. For the sensitivity analysis, see **Supplementary Table S3**.

**Table 2:** Egger’s test results.

Dimensions	Egger’s test		Bias
	T-value	*P*-value	
Age	0.7114,	0.4931	No
Diabetes duration	4.4954	0.0020	Yes
Educational attainment	-1.0545	0.3083	No
HbA1c	2.0632	0.0597	No

### 3.4 Comprehensive results analysis

#### 3.4.1 Demographic and clinical characteristics

**Figure 3:**
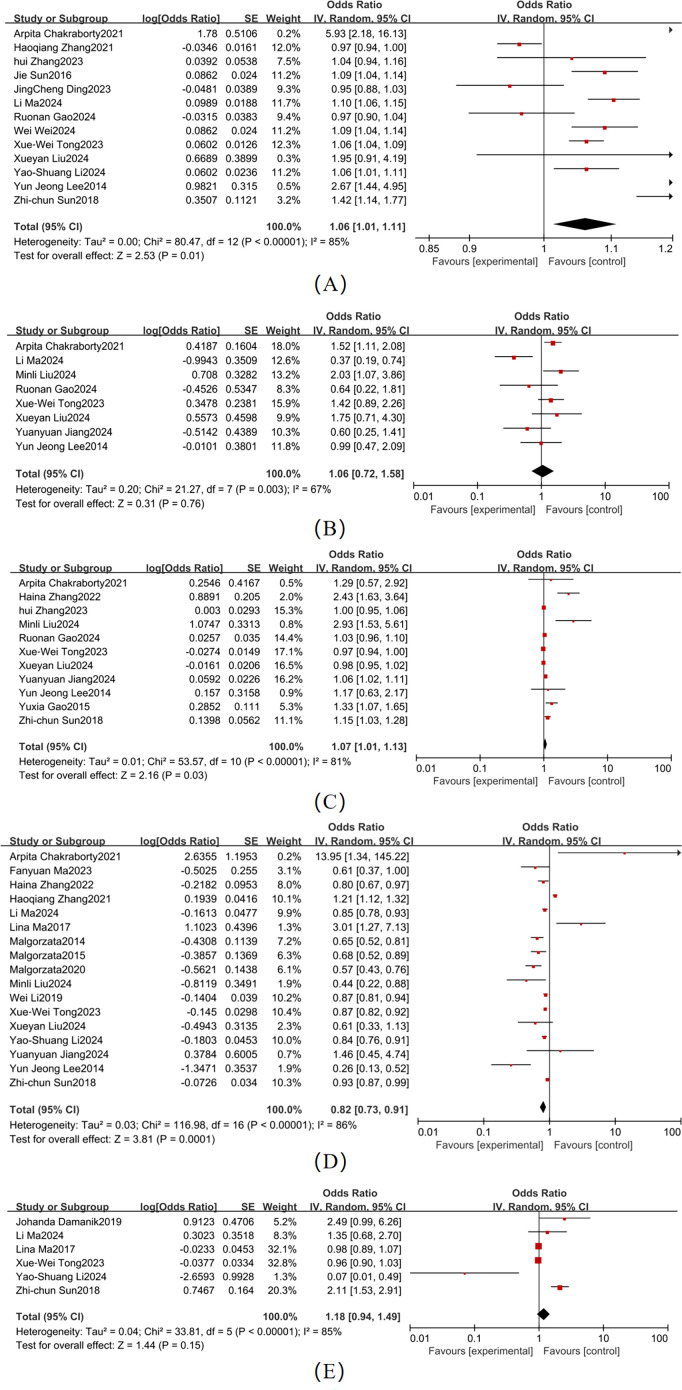
Forest map of demographic and clinical characteristics **(A)** Age; **(B)** Sex; **(C)** Duration; **(D)** Level of education; **(E)** BMI.

Significant heterogeneity was observed across studies for age, sex, diabetes duration, educational attainment, and BMI (**Figure 3**). Pooled effect sizes demonstrated the following outcomes: Age (13 studies; χ² = 80.47, *P* < 0.001, I² = 85%): OR = 1.06 (95% CI: 1.01–1.11, *P* = 0.01); Female sex (8 studies; χ² = 21.27, *P* = 0.03): OR = 1.23 (95% CI: 1.00–1.50, *P* = 0.05), I² = 67%,; Diabetes duration (11 studies; χ² = 53.57, *P* < 0.001, I² = 81%): OR = 1.07 (95% CI: 1.01–1.13, *P* = 0.03); and Educational attainment (17 studies; χ² = 116.98, *P* < 0.001, I² = 86%): OR = 0.82 (95% CI: 0.73–0.91, *P* = 0.0001). Forest plots for these factors showed 95% CI that did not overlap with the null line, indicating statistically significant associations with T2DM-MCI comorbidity. In contrast, BMI (6 studies; χ² = 33.81, *P* < 0.001, I² = 85%) showed a non-significant pooled effect size (OR = 1.18, 95% CI: 0.94–1.49, *P* = 0.15), with the CI ranges overlapping the null line. For age (I² = 85%), sex (I² = 67%), diabetes duration (I² = 81%), educational attainment (I² = 86%), and BMI (I² = 85%), the heterogeneity was greater than 50%, so a random-effects model was used for all.

**Figure 4:**
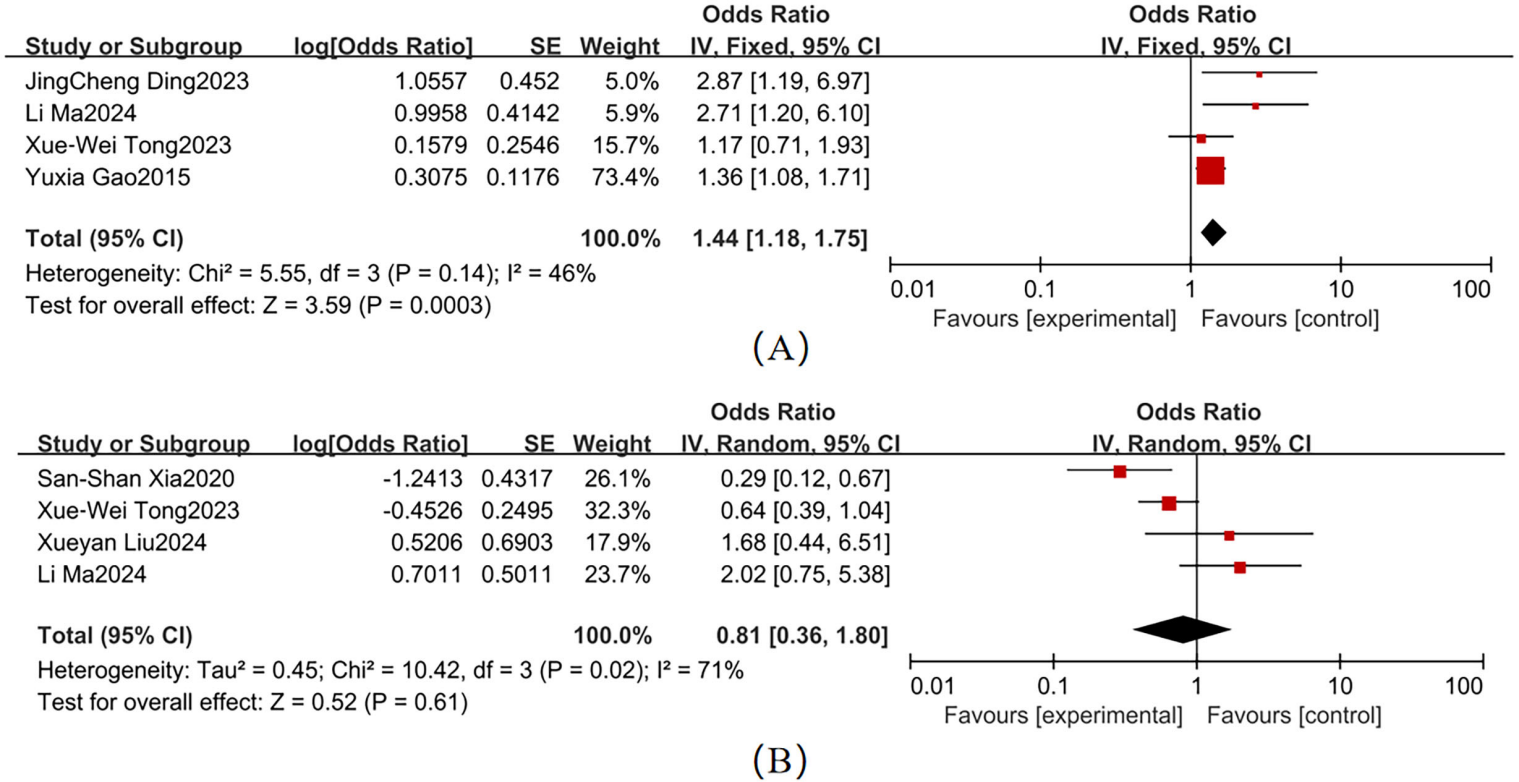
Lifestyle Forest map **(A)** Smoking; **(B)** Alcohol Consumption.

Moderate heterogeneity was observed across studies for smoking and alcohol consumption (**Figure 4**). Key findings were as follows: Smoking (4 studies; χ² = 5.55, *P* = 0.14, I² = 46%): Pooled OR = 1.44 (95% CI: 1.18–1.75, *P* = 0.003), When I² <50%, a fixed-effects model was used. Forest plot analysis showed non-overlapping 95% CI with the null line, indicating a statistically significant association with T2DM-MCI comorbidity. Alcohol consumption (4 studies; χ² = 10.42, *P* = 0.02, I² = 71%): Pooled OR = 0.81 (95% CI: 0.36–1.80, *P* = 0.61), When I² >50%, a fixed-effects model was used. The 95% CI ranges overlapped the null line, suggesting no statistically significant association.

#### 3.4.2 Comorbidity management

**Figure 5:**
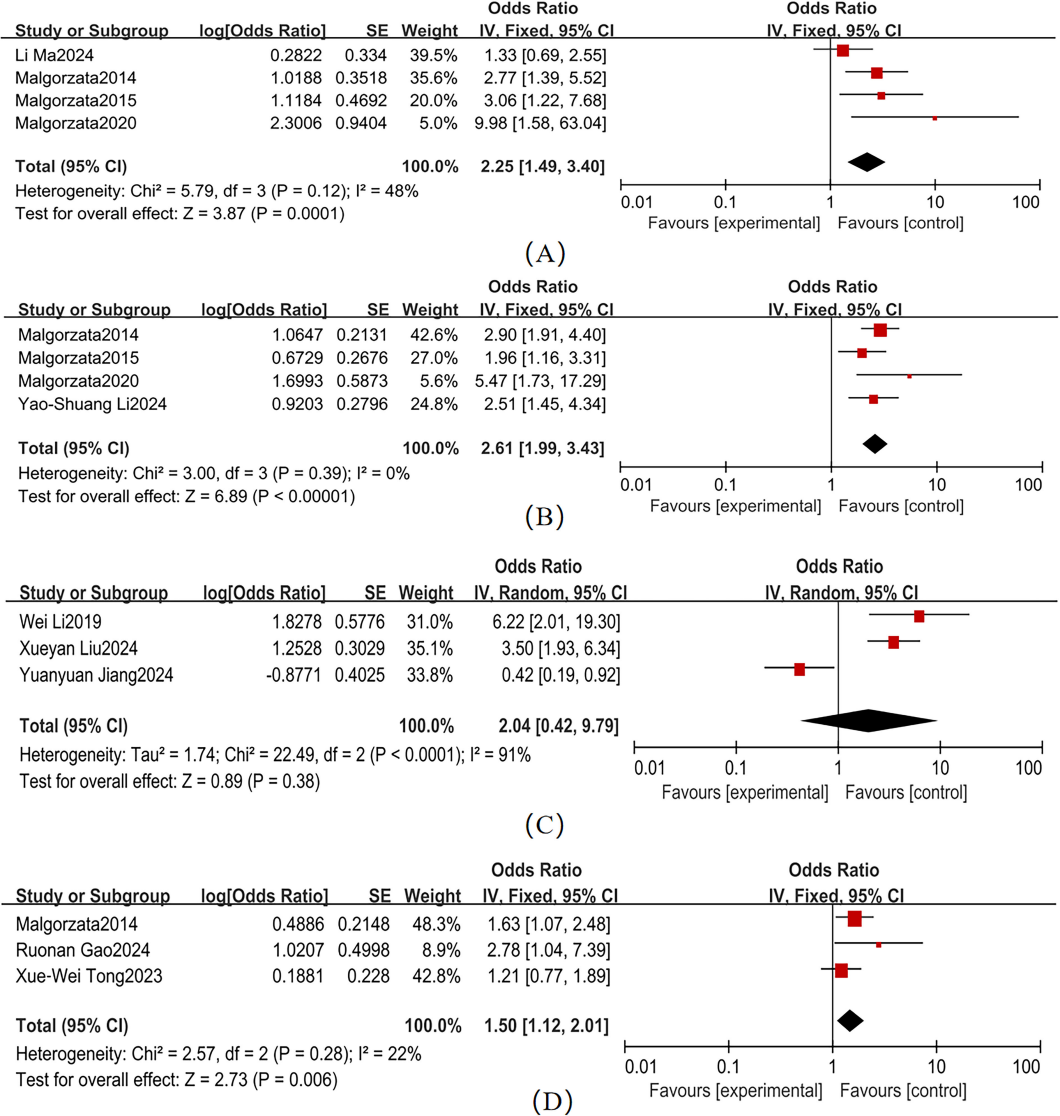
Comorbidity management forest map **(A)** Hypertension; **(B)** CVD; **(C)** Depression; **(D)** DR.

Variable heterogeneity was observed across studies for hypertension, CVD, and DR (**Figure 5**). The pooled effect sizes were as follows: hypertension (4 studies; χ² = 5.79, *P* = 0.12, I² = 48%): OR = 2.25 (95% CI: 1.49–3.40, *P* < 0.001); CVD (4 studies; χ² = 3.00, *P* = 0.39, I² = 0%): OR = 2.61 (95% CI: 1.99–3.43, *P* < 0.001); and DR (3 studies; χ² = 2.57, *P* = 0.28, I² = 22%): OR = 1.50 (95% CI: 1.12–2.01, *P* = 0.006). Forest plots for these comorbidities showed non-overlapping 95% CI with the null line, indicating statistically significant associations with T2DM-MCI comorbidity. In contrast, depression (3 studies; χ² = 22.49, *P* < 0.001, I² = 91%) exhibited a non-significant pooled effect size (OR = 2.04, 95% CI: 0.42–9.79, *P* = 0.38), despite its 95% CI range overlapping the null line. Hypertension (I² = 48%), CVD (I² = 0%), and DR (I² = 22%) had I² < 50%, so a fixed-effects model was used. Depression (I² = 91%) had I² > 50%, so a random-effects model was used.

#### 3.4.3 Biochemical indicators

**Figure 6:**
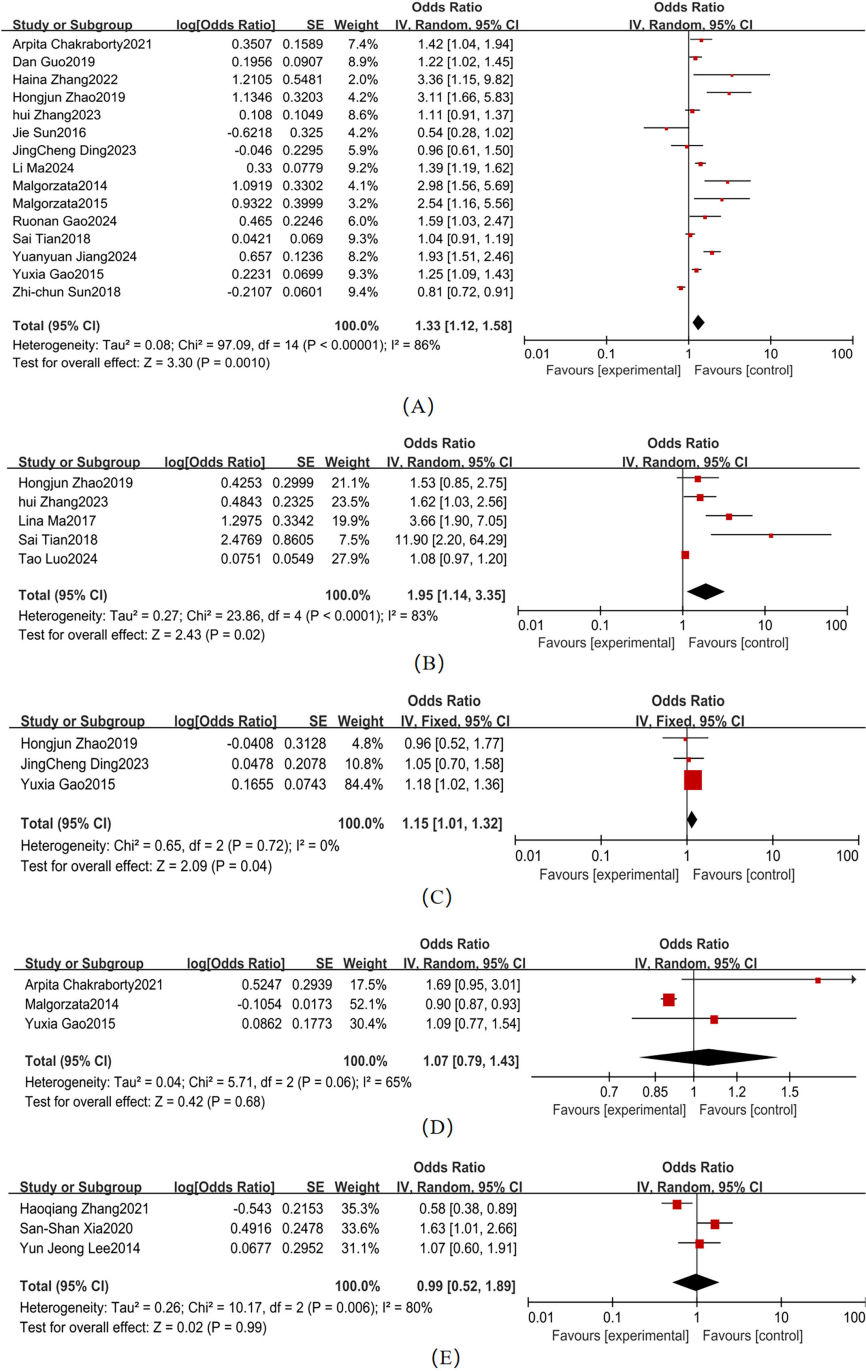
Biochemical Indicators Forest map **(A)** HbA1c; **(B)** HOMA-IR; **(C)** FPG; **(D)** HDL; **(E)** LDL-C (2).

Substantial heterogeneity was observed across studies for HbA1c, HOMA-IR, FPG, and HS-CRP (**Figure 6**). Pooled effect sizes demonstrated: HbA1c (15 studies; χ² = 97.09, *P <*0.001, I² = 86%): OR = 1.33 (95% CI: 1.12–1.58, *P* = 0.001)、HOMA-IR(5 studies; χ² = 23.86, *P* < 0.001, I² = 83%): OR = 1.95 (95% CI: 1.14–3.35, *P* = 0.02)、FPG (3 studies; χ² = 0.65, *P* = 0.06, I² = 0%): OR = 1.15 (95% CI: 1.01–1.32, = 0.04); and HS-CRP (3 studies; χ² = 1.65, *P* = 0.44, I² = 0%): OR=2.85 (95% CI: 2.09–3.89, *P* < 0.001). Forest plots showed non-overlapping 95% CI with the null line for these parameters, confirming statistically significant associations with T2DM-MCI comorbidity. HDL (3 studies; χ² = 5.71, *P* = 0.06, I² = 65%) and LDL-C (3 studies; χ² = 10.17, *P* = 0.006, I² = 80%) exhibited non-significant pooled effect sizes: HDL: OR = 1.07 (95% CI: 0.79–1.43, *P* = 0.68); and LDL-C: OR = 0.99 (95% CI: 0.52–1.89, *P* = 0.99). The overlapping 95% CI with the null line indicated no statistically significant associations for these lipid parameters. HbA1c (I² = 86%), HOMA-IR (I² = 83%), HDL (I² = 65%), and LDL-C (I² = 80%) had I² > 50%, so a random-effects model was used. FPG (I² = 0%) and HS-CRP (I² = 0%) had I² < 50%, so a fixed-effects model was used.

#### 3.4.4 Subgroup analysis

**Figure 7:**
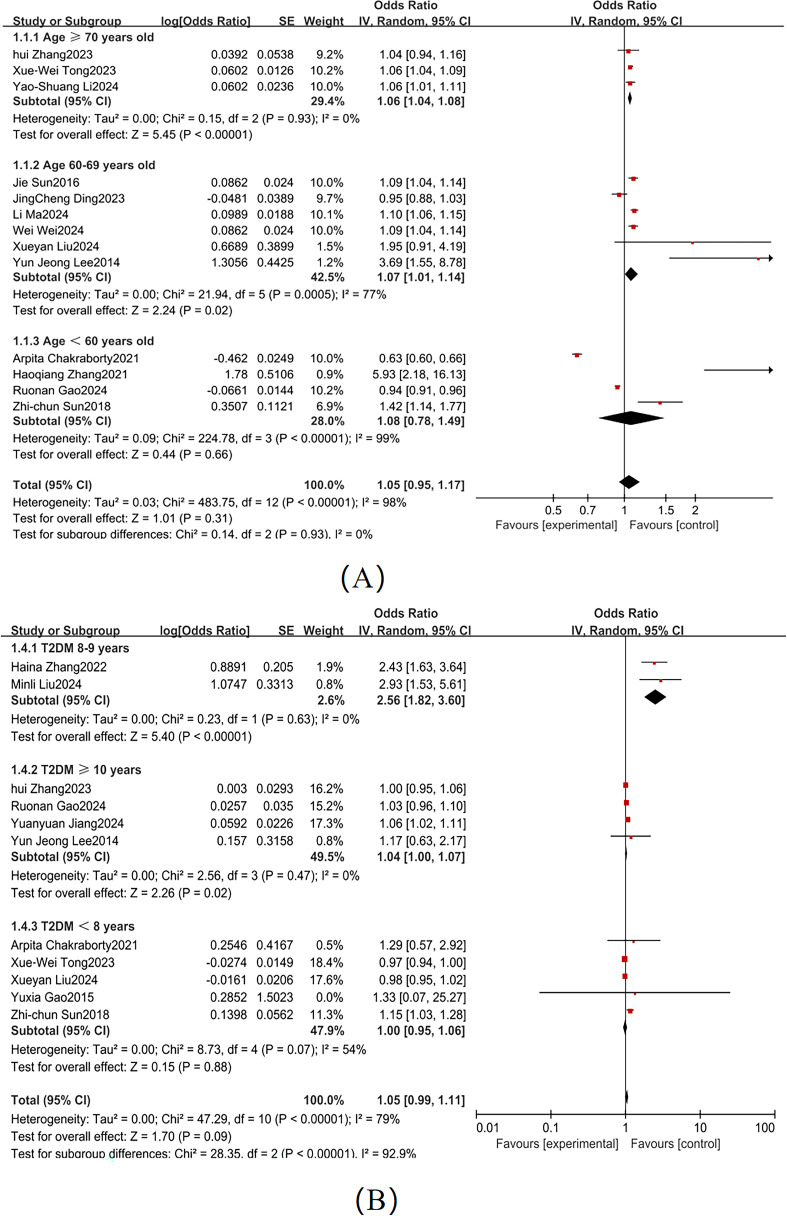
Subgroup analysis **(A)** Age; **(B)** Duration.

According to the American Diabetes Association guidelines, diabetes patients aged ≥70 are generally at higher risk for complications, particularly in terms of cognitive function and cardiovascular health (62). Based on the dataset, we divided age into three categories: ≥70 years, 60–69 years, and <60 years. Significant heterogeneity was observed across these subgroups (I² = 74%; **Figure 7A**), so a random-effects model was used. The American Diabetes Association points out that patients with a diabetes duration of ≥10 years have a significantly increased risk of cognitive decline (62). Based on the dataset, we categorized diabetes duration into ≥10 years, 8–9 years, and <8 years, with substantial heterogeneity between subgroups (I² = 81%; **Figure 7B**). Subgroup analyses identified the following independent risk factors for MCI development in T2DM patients: advanced age (≥ 70 years: OR = 1.06, 95% CI: 1.04-1.08, *P* < 0.001); prolonged diabetes duration (≥10 years: OR = 1.04, 95% CI: 1.01-1.07, *P* = 0.02) as independent risk factors for MCI development in T2DM patients.

**Figure 8:**
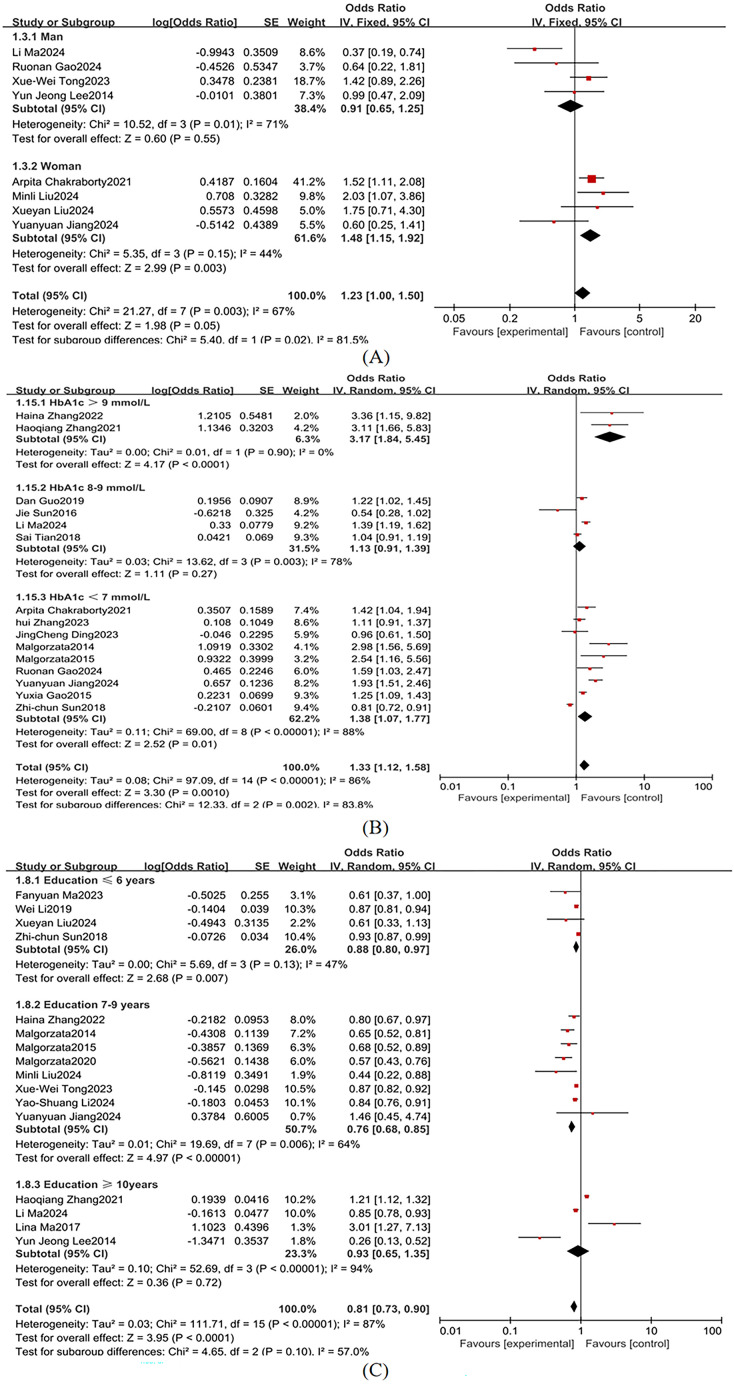
Subgroup analysis **(A)** Sex; **(B)** HbA1C; **(C)** Educational attainment.

Sex was stratified into male and female subgroups, demonstrating significant between-group heterogeneity in effect sizes (I² = 81.5%; **Figure 8A**), so a random-effects model was used. According to the American Diabetes Association guidelines, an HbA1c level above 9%, typically indicates poor diabetes control, with a higher risk of complications. An HbA1c level <7% is the treatment goal for most diabetes patients to reduce the risk of diabetes-related complications (62). Therefore, we categorized HbA1c into >9%, 8-9%, and <7%, with substantial heterogeneity across subgroups (I² = 86%; **Figure 8B**), so a random-effects model was used. Educational attainment was stratified into ≤6 years, 7–9 years, and ≥10 years, demonstrating marked heterogeneity (I² = 87%; **Figure 8C**), so a random-effects model was used. Key subgroup analyses identified the following independent risk factors for MCI in T2DM populations: female sex: OR = 1.48 (95% CI: 1.15-1.91, *P* = 0.003); HbA1c >9%: OR = 3.17 (95% CI: 2.35-4.28, *P <*0.001); educational attainment ≤ 6 years: OR = 1.29 (95% CI: 0.80-0.97, *P* = 0.007) as independent risk factors for MCI in T2DM populations.

## 4 Discussion

This study systematically evaluated the influencing factors of MCI comorbidity in patients with T2DM through meta-analysis, revealing multidimensional interactions among demographic/clinical characteristics, lifestyle factors, disease management, and biochemical indicators. The results indicate that age and educational attainment exhibit the highest predictive weights among demographic and clinical characteristics. For lifestyle factors, smoking demonstrates the strongest association with T2DM-MCI comorbidity. In disease management, CVD and hypertension emerge as core risk factors. Among biochemical indicators, HbA1c is identified as the most significant factor, followed by the heavily weighted HOMA-IR.

### 4.1 Demographic and clinical determinants of T2DM-MCI comorbidity

Subgroup analysis in this study demonstrated that T2DM patients aged ≥ 70 years face an elevated MCI risk (OR = 1.06, 95% CI: 1.04-1.08, *P* < 0.001), while those with diabetes aged 60–69 years showed a higher risk (OR = 1.07, 95%CI:1.01-1.14, *P* = 0.005). These findings align with Sun et al. (63), confirming age as a non-modifiable risk factor for MCI. Potential mechanisms include age-related neurodegeneration (e.g., neuronal loss, reduced synaptic density, and impaired cerebral energy metabolism) (64), compounded by elevated advanced glycation end products (AGEs) and oxidative stress in elderly T2DM patients (65), which synergistically accelerate vascular dysfunction and cognitive deterioration (66).

Female sex was identified as a significant risk factor for MCI in T2DM patients (OR = 1.48 vs. 0.91 in men), consistent with You et al. (67). While gender differences remain debated (68, 69), emerging evidence suggests that the postmenopausal decline in estrogen may attenuate neuroprotective pathways (68, 70). Additionally, sex-specific disparities in cardiovascular risks profiles (71), adiposity distribution patterns (72), and chronic inflammatory states (73) likely contribute to this association. Although these mechanisms are not fully understood, existing studies support the significant role of sex in diabetes-related cognitive impairment.

The present study highlights a strong relationship between diabetes duration and MCI risk. Subgroup analyses revealed significantly elevated risks in patients with longer disease duration: those with ≥ 10 years of diabetes exhibited an adjusted OR of 1.04 (95%CI:1.01-1.07, *P* = 0.02), while those with 8–9 years diabetes showed a higher risk (OR = 2.56, 95%CI:2.13-3.08, *P* < 0.001). These findings suggest that prolonged hyperglycemia accelerates cognitive dysfunction through cumulative metabolic insults. Longitudinal studies indicate that patients with ≥ 20 years of diabetes have a 3.32-fold increased risk of information processing deficits, a 1.72-fold risk of immediate recall impairment, and a 1.76-fold risk of executive dysfunction compared to those with shorter disease duration (74). Mechanistically, chronic hyperglycemia drives insulin resistance, intermittent hypoglycemia, and microvascular complications (75). Furthermore, extended disease duration may induce structural and functional brain changes (e.g. accelerating cerebral atrophy and reduced synaptic density) and functional neurodegeneration (76), which collectively contribute to cognitive decline.

Low educational attainment (≤ 6 years) was associated with an increased risk of MCI (OR = 1.29, 95% CI: 0.80–0.97, P = 0.007), supporting the cognitive reserve hypothesis (77). Higher educational attainment may enhance neural plasticity and compensatory mechanisms, potentially delaying cognitive decline. Additionally, greater education may optimize neural network efficiency, helping maintain cognitive resilience despite chronic metabolic conditions such as diabetes (36).

No significant association was found between BMI and MCI risk in patients with T2DM (OR = 1.18, 95% CI: 0.94–1.49, *P* = 0.15). Although elevated BMI is associated with insulin resistance (78), adiposity-related inflammation (79), and cardiovascular risks (80)—factors that may indirectly impair cerebral metabolism and cognition— BMI was not independently associated with MCI in this cohort. This finding may reflect interactions between BMI and confounding variables (e.g., age, glycemic control, sex). Future research should clarify BMI’s role through stratified analyses and longitudinal studies.

### 4.2 Lifestyle factors and T2DM-MCI comorbidity

It was demonstrated that smoking increases the MCI risk in patients with T2DM (OR = 1.44, 95% CI: 1.18–1.75, *P* = 0.0003). These findings align with previous studies (81, 82), confirming smoking as a critical risk factor for T2DM-MCI comorbidity. For instance, Hagger-Johnson et al. (83) reported accelerated cognitive decline in middle-aged and elderly smokers, while Xia et al. (84) identified an inverse correlation between smoking intensity and serum brain-derived neurotrophic factor levels—a key mediator of neurogenesis and synaptic plasticity. Mechanistically, nicotine may impair cognition through interactions with nicotinic acetylcholine receptor subunits (e.g., α4, β2, and α7) [ (85). Although precise pathways require further elucidation, the robust association between smoking and cognitive deterioration is well-established.

In contrast, alcohol consumption showed no significant association with T2DM-MCI comorbidity in this study (OR = 0.81, 95% CI: 0.36–1.80, *P* = 0.61). However, existing evidence (86) suggests a complex dose-response dynamic, including U-shaped or J-shaped relationships. A Finnish cohort study of 1,464 adults aged 65–79 years found that midlife heavy drinking patterns significantly increased MCI risk (OR = 5.08, *P* = 0.020) (87), while another study reported elevated cognitive decline risks in both heavy drinkers (OR = 1.44, 95% CI: 1.02–2.10) and abstainers (OR = 1.94, 95% CI: 1.10–3.44) compared to moderate drinkers (88). The non-significant association observed here may reflect limitations of the cross-sectional design or population heterogeneity, necessitating longitudinal studies to clarify the role of alcohol in T2DM-related cognitive dysfunction.

### 4.3 Comorbidity management and T2DM-MCI comorbidity

Hypertension was identified as a significant risk factor for MCI in patients with T2DM (OR = 2.25, 95% CI: 1.49–3.40, *P* < 0.001), consistent with prior research (89, 90). Chronic hypertension induces structural and functional cerebrovascular damage through ischemic white matter injury and microvascular pathology, reducing cerebral blood flow and accelerating cognitive decline (91). Similarly, CVD significantly elevates MCI risk (OR = 2.61, 95% CI: 1.99–3.43, *P* < 0.001), corroborating Xie et al. (92). Post-stroke cerebrovascular injuries—particularly those involving extracranial carotid or intracranial vascular lesions (93)—are strongly associated with cognitive impairment in diabetic populations. Notably, diabetic stroke survivors with larger infarct volumes exhibit pronounced cognitive deficits, substantially increasing post-stroke cognitive impairment (PSCI) risk (94). These findings underscore the need for intensified CVD management in T2DM patients to mitigate cognitive deterioration.

Interestingly, depression showed no significant association with MCI risk (OR = 2.04, 95% CI: 0.42–9.79, *P* = 0.38), contrasting with Carr et al. (95) and Chow et al. (96). While depression-related neurodegeneration in brain regions such as the hippocampus and prefrontal cortex may drive cognitive dysfunction (97), confounding factors [e.g. glycemic control (98), systemic inflammation (99)] and regional population differences likely explain this discrepancy.

DR emerged as a significant MCI predictor (OR = 1.50, 95% CI: 1.12–2.01, *P* = 0.006), corroborating Gorska-Ciebiada et al. (45). As a microvascular complication, DR shares pathophysiological mechanisms with cerebral microangiopathy [e.g. chronic hyperglycemia-induced endothelial dysfunction (100)], suggesting its potential role as a biomarker for concurrent brain microvascular damage (101). Proactive DR screening and management may thus help reduce MCI risk in T2DM patients.

### 4.4 Biochemical indicators and T2DM-MCI comorbidity

It was found that HbA1c >9% significantly elevates MCI risk in patients with T2DM (OR = 1.33, *P* = 0.001), with residual risks persisting even within the conventional glycemic target range (HbA1c <7%: OR = 1.38, *P* = 0.002). Chronic hyperglycemia impairs glial cell function, inducing cerebrovascular pathology and neuronal damage that ultimately compromise cognition (102). Zheng et al. (103) reported that each 1 mmol/mol increase in HbA1c exacerbates declines in cognitive, memory, and executive functions. Hyperglycemia-driven cognitive deterioration involves multiple mechanisms, including disrupted neurotransmitter metabolism (104), aggravated oxidative stress (105), and altered neuronal energy homeostasis (106), solidifying HbA1c’s role as a critical biomarker for T2DM-MCI comorbidity.

Elevated HOMA-IR was independently associated with MCI risk (OR = 1.95, 95% CI: 1.14–3.35, *P* = 0.02). As a surrogate marker of insulin resistance, HOMA-IR reflects compensatory hyperinsulinemia, which is closely linked to cognitive decline. Kim et al. (107) identified strong associations between hyperinsulinemia and impairments in memory and executive function. Pharmacological interventions improving insulin sensitivity have shown potential to enhance memory performance (108), suggesting therapeutic relevance. Although insulin resistance-MCI relationships are empirically supported (108, 109), underlying mechanisms remain incompletely elucidated, necessitating further investigation of HOMA-IR’s predictive utility.

A potential association between elevated FPG and MCI risk in T2DM patients was also identified (OR = 1.15, 95% CI: 1.01–1.32, *P* = 0.04). Comparative analysis revealed lower FPG levels in non-MCI groups, suggesting that sustained hyperglycemia may contribute to cognitive dysfunction. Chronic hyperglycemia not only exacerbates microvascular complications but also induces long-term neurological detriment, oxidative stress, and blood-brain barrier disruption (110, 111). Thus, FPG serves dual roles as a glycemic control biomarker and a predictor of cognitive decline in T2DM.

HDL levels showed no significant association with MCI risk in T2DM patients (OR = 1.07, 95% CI: 0.79–1.43, *P* = 0.68), diverging from studies suggesting HDL’s neuroprotective effects via anti-inflammatory and antioxidant pathways (112, 113). Moderate heterogeneity across studies (I² = 65%, P = 0.06) may stem from methodological variations in HDL measurement or population characteristics.

No significant association was found between LDL-C levels and MCI risk in the current study (OR = 0.99, 95% CI: 0.52–1.89, *P* = 0.99). Despite proposed U-shaped relationships between LDL-C and cognitive function (114), significant heterogeneity (I² = 80%, *P* = 0.006) suggests confounding effects from diabetes control status or inflammatory mediators, necessitating further investigation into these complex interactions.

Finally, elevated HS-CRP was strongly associated with MCI risk (OR = 2.85, 95% CI: 2.09–3.89, *P <*0.001). As a sensitive inflammatory biomarker (115), elevated HS-CRP reflects systemic inflammation that synergizes with oxidative stress to amplify free radical generation, damaging cellular membranes, DNA, and neuronal function (116, 117). These findings highlight HS-CRP’s dual roles as an inflammatory marker and a predictor of early cognitive decline in T2DM.

## 5 Limitations

This study has several limitations. First, the generalizability of the findings may be constrained by geographical and population homogeneity, as most of the included studies focused on Chinese populations, with limited representation of other ethnic or regional groups. Second, methodological heterogeneity—including variability in study designs (e.g., cross-sectional vs. cohort), sample sizes, and quality—likely contributed to substantial heterogeneity, which introduces a cautious interpretation of pooled estimates. Third, the predominance of cross-sectional designs (which constitute a high proportion of the included studies) precludes causal inference or longitudinal trajectory analysis. To address these limitations, future research should prioritize large-scale, multi-center cohort studies with extended follow-up periods to validate the identified risk factors. Additionally, a systematic investigation of unexplored confounders (e.g., genetic predisposition, lifestyle interactions) and mechanistic pathways is warranted to comprehensively elucidate T2DM-MCI pathophysiology.

## 6 Conclusions

This meta-analysis identified advanced age (≥ 60 years), female sex, prolonged diabetes duration (8–9 years), elevated HbA1c (> 9%), and low educational attainment (≤ 6 years) as significant independent predictors of MCI in patients with T2DM, demonstrating clear dose-response relationships. Smoking, hypertension, CVD, insulin resistance (as measured by the HOMA-IR), FPG, and HS-CRP were also significantly associated with increased MCI risk.

These findings underscore the need for integrated clinical strategies. Regular cognitive assessments should target high-risk subgroups, including elderly patients with long-standing diabetes (8–9 years), females with poor glycemic control (HbA1c > 9%), and individuals with vascular comorbidities. Glycemic management aiming for HbA1c < 7% may offer cognitive protection, while population with limited education warrant tailored health literacy interventions to mitigate self-management barriers. Multidisciplinary collaboration across endocrinology, neurology, and ophthalmology is critical for patients with microvascular complications, such as diabetic retinopathy.

This study advances the understanding of T2DM-MCI determinants but highlights key research gaps. Future investigations should prioritize longitudinal designs to establish causality, validate biomarkers across diverse populations, and explore mechanistic interactions between metabolic dysregulation and neurodegeneration. Such efforts will be essential for developing precision prevention frameworks against diabetic cognitive decline. Since the results of this study are mainly based on Chinese populations, their applicability to other regions or races may be limited. Follow-up research should include more diverse samples to enhance the external validity of the research.

## Data Availability

The original contributions presented in the study are included in the article/**Supplementary Material**. Further inquiries can be directed to the corresponding authors.
