# Supplementary material for: Risk factors for mild cognitive impairment in type 2 diabetes: a systematic review and meta-analysis
Source: Front Endocrinol (Lausanne). 2025 Jun 16;16:1617248. doi: 10.3389/fendo.2025.1617248 (PMC12206637; doi:10.3389/fendo.2025.1617248)
Supplement: Supplementary file 1 [file Table1.docx]

Supplementary Material

Table S1 Newcastle–Ottawa scale quality assessment results

| **Studies** | **Newcastle–Ottawa scale** | | | |
| --- | --- | --- | --- | --- |
|  | Selection (0–4) | Comparability (0–2) | Outcome/Exposure (0–3) | Total |
| Xueyan Liu 2024 | 3 | 1 | 2 | 6 |
| Yuanyuan Jiang 2024 | 3 | 1 | 3 | 7 |
| Wei Wei 2024 | 3 | 2 | 3 | 8 |
| Tao Luo 2024 | 3 | 1 | 3 | 7 |
| Minli Liu 2024 | 3 | 2 | 3 | 8 |
| Miaoyan Zheng 2019 | 3 | 2 | 3 | 8 |
| Fanyuan Ma 2023 | 4 | 2 | 3 | 9 |
| Ruonan Gao 2024 | 3 | 2 | 3 | 8 |
| Jingcheng Ding 2023 | 3 | 1 | 3 | 7 |

Table S2 Joanna Briggs Institute quality assessment results

| Studies | **Joanna Briggs Institute** | | | | | | | | Total |
| --- | --- | --- | --- | --- | --- | --- | --- | --- | --- |
|  | Inclusion criteria are clearly defined | The participants and the environment are described in detail | Exposure measured in a valid and reliable way | The criterion used to measure the condition was objective | Confounding factors were identified | Strategies for dealing with confounding factors | Results measured in a valid and reliable way | Appropriate statistical analysis was used |  |
| Malgorzata Gorska-Ciebiada 2014 | Y | Y | C | Y | N | N | Y | Y | 5 |
| San-Shan Xia 2020 | Y | Y | N | C | Y | N | Y | Y | 5 |
| Malgorzata Gorska-Ciebiada 2016 | Y | Y | C | Y | N | N | Y | Y | 5 |
| Arpita Chakraborty 2021 | C | Y | Y | Y | N | N | Y | Y | 5 |
| Dan Guo 2019 | Y | Y | Y | Y | C | C | Y | Y | 6 |
| Yun Jeong Lee 2014 | C | Y | Y | Y | Y | N | Y | Y | 6 |
| Hui Zhang 2023 | C | Y | Y | Y | Y | C | Y | Y | 6 |
| Sai Tian 2018 | Y | Y | Y | Y | Y | N | Y | Y | 7 |
| Hongjun Zhao 2019 | Y | Y | N | C | Y | N | Y | Y | 5 |
| Haoqiang Zhang 2021 | Y | Y | Y | Y | N | C | Y | Y | 6 |
| Wei Li 2019 | Y | Y | Y | Y | Y | N | Y | Y | 7 |
| Zhichun Sun 2018 | Y | Y | Y | Y | N | C | Y | Y | 6 |
| Yaoshuang Li 2024 | C | N | Y | Y | Y | C | Y | Y | 5 |
| Jie Sun 2016 | Y | Y | C | Y | N | N | Y | Y | 5 |
| Li Ma 2024 | Y | Y | Y | Y | Y | N | Y | Y | 7 |
| Xuewei Tong 2023 | Y | Y | Y | Y | N | C | Y | Y | 6 |
| Haina Zhang 2023 | C | Y | Y | Y | N | N | Y | Y | 5 |
| Malgorzata Gorska-Ciebiada 2020 | Y | Y | C | Y | N | N | Y | Y | 5 |
| Johanda Damanik 2019 | C | Y | Y | Y | N | N | Y | Y | 5 |
| Lina Ma 2017 | Y | Y | Y | Y | N | C | Y | Y | 6 |
| Yuxia Gao 2016 | Y | Y | Y | Y | Y | N | Y | Y | 7 |

Table S3 Sensitivity analysis results

| Risk Factors | Effect model OR [95%CI] | | Stability |
| --- | --- | --- | --- |
|  | Fixed effect model | Random Effect model |  |
| Age | 1.05 [1.03, 1.06] | 1.06 [1.01, 1.11] | Stable |
| Sex | 1.23 [1.00, 1.50] | 1.06 [0.72, 1.58] | Unstable |
| Diabetes duration | 1.01 [0.99, 1.03] | 1.07 [1.01, 1.13] | Stable |
| Depression | 1.98 [1.28, 3.07] | 2.04 [0.42, 9.79] | Unstable |
| Alcohol | 0.69 [0.48, 1.01] | 0.81 [0.36, 1.80] | Unstable |
| Smoking | \| 1.44 [1.18, 1.75] \| \| --- \| | 1.59 [1.11, 2.28] | Stable |
| Educational attainment | 0.90 [0.87, 0.92] | \| 0.82 [0.73, 0.91] \| \| --- \| | Stable |
| BMI | \| 0.99 [0.94, 1.04] \| \| --- \| | 1.18 [0.94, 1.49] | Unstable |
| CVD | 2.61 [1.99, 3.43] | 2.61 [1.99, 3.43] | Stable |
| Hypertension | 2.25 [1.49, 3.40] | 2.49 [1.35, 4.62] | Stable |
| Hs-CRP | 2.85 [2.09, 3.89] | \| 2.85 [2.09, 3.89] \| \| --- \| | Stable |
| LDL-C | 0.94 [0.71, 1.25] | 0.99 [0.52, 1.89] | Stable |
| HbA1c | 1.15 [1.09, 1.22] | 1.33 [1.12, 1.58] | Stable |
| HOMA-IR | 1.16 [1.04, 1.28] | 1.95 [1.14, 3.35] | Unstable |
| FBG | 1.15 [1.01, 1.32] | 1.15 [1.01, 1.32] | Stable |
| HDL | 0.90 [0.87, 0.93] | 1.07 [0.79, 1.43] | Unstable |
| DR | 1.50 [1.12, 2.01] | 1.53 [1.08, 2.16] | Stable |
